# Supplementary material for: Identification of Long-Distance Transport Signal Molecules Associated with Plant Maturity in Tetraploid Cultivated Potatoes (Solanum tuberosum L.)
Source: Plants (Basel). 2022 Jun 28;11(13):1707. doi: 10.3390/plants11131707 (PMC9268856; doi:10.3390/plants11131707)
Supplement: Supplementary file 1 [file plants-11-01707-s001.zip › Figure S1. Venn diagram of DEGs related to the potato plant early-maturity (a) and late-maturity (b) traits..pdf]

Figure S1. Venn diagram of DEGs related to the potato plant early-maturity (a) and late-maturity (b) traits.

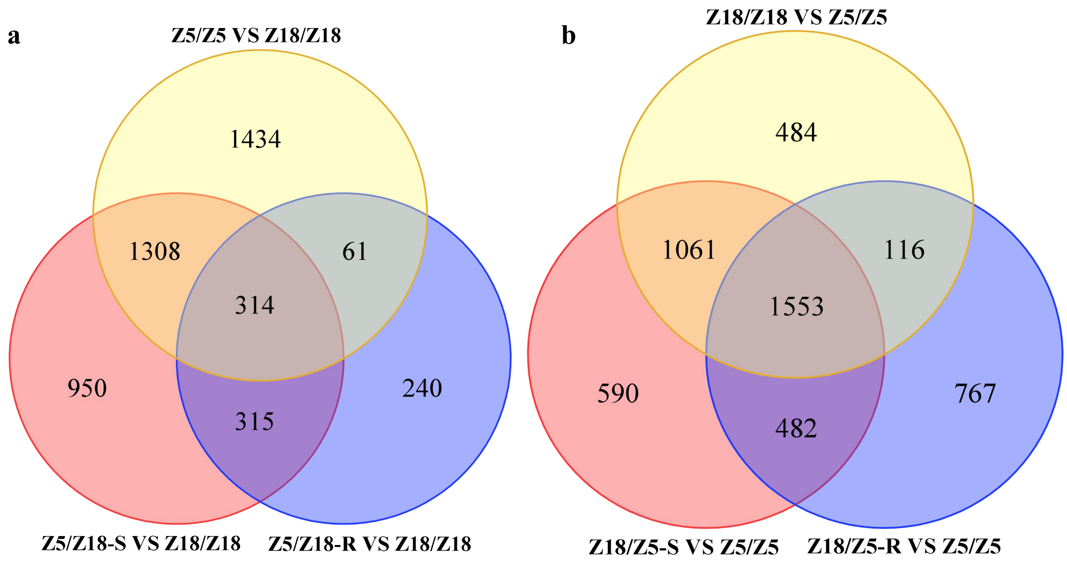

Figure S1. Venn diagram of DEGs related to the potato plant early-maturity (a) and late-maturity (b) traits. Z5/Z18-S, Z5 stem of early-maturing cultivar Z5 grafted onto late-maturing cultivar Z18. Z5/Z18-R, Z18 stem of early-maturing cultivar Z5 grafted onto late-maturing cultivar Z18. Z18/Z5-S, Z18 stem of late-maturing cultivar Z18 grafted onto early-maturing cultivar Z5. Z18/Z5-R, Z5 stem of late-maturing cultivar Z18 grafted onto early-maturing cultivar Z5. Z5/Z5, Z5 stem of early-maturing cultivar Z5 was self-grafted. 18/Z18, Z18 stem of late-maturing cultivar Z18 was self-grafted.
