# Supplementary material for: Identification of Long-Distance Transport Signal Molecules Associated with Plant Maturity in Tetraploid Cultivated Potatoes (Solanum tuberosum L.)
Source: Plants (Basel). 2022 Jun 28;11(13):1707. doi: 10.3390/plants11131707 (PMC9268856; doi:10.3390/plants11131707)
Supplement: Supplementary file 1 [file plants-11-01707-s001.zip › Figure S4. Venn diagram of the differential metabolites associated with the potato plant early-maturity(a) and late-maturity(b).pdf]

Figure S4. Venn diagram of the differential metabolites associated with the potato plant early-maturity(a) and late-maturity(b)

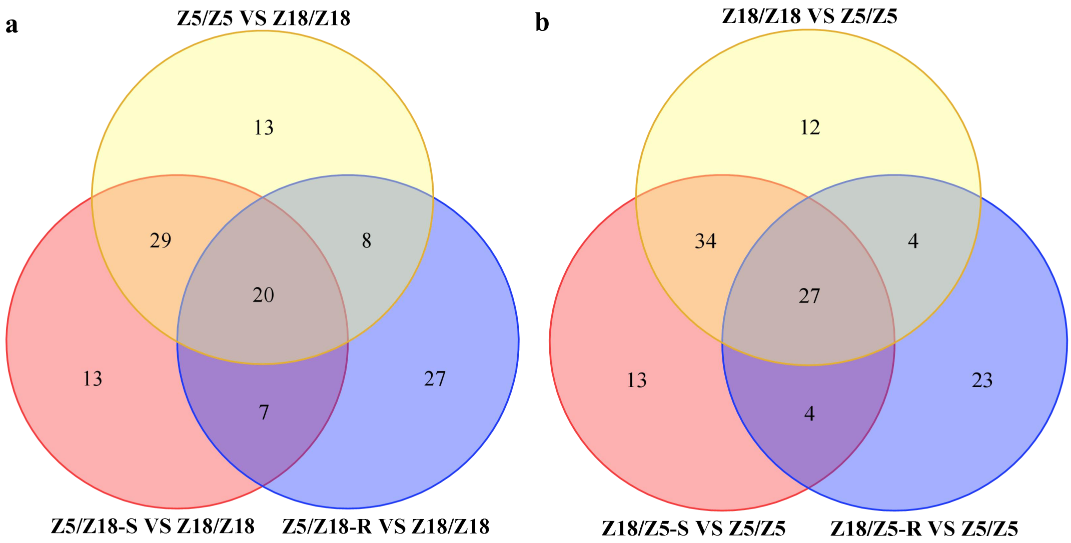

Figure S4. Venn diagram of the differential metabolites associated with the potato plant early-maturity(a) and late-maturity(b). **Z5/Z18-S:** Z5 stems of early-maturing cultivar Z5 grafted onto late-maturing cultivar Z18. **Z5/Z18-R:** Z18 stems of early-maturing cultivar Z5 grafted onto late-maturing cultivar Z18. **Z18/Z5-S:** Z18 stems of late-maturing cultivar Z18 grafted onto early-maturing cultivar Z5. **Z18/Z5-R:** Z5 stems of late-maturing cultivar Z18 grafted onto early-maturing cultivar Z5. **Z5/Z5:** Z5 stems of early-maturing cultivar Z5 was self-grafted. **Z18/Z18:** Z18 stemS of late-maturing cultivar Z18 was self-grafted.
