# Supplementary material for: Identification of Long-Distance Transport Signal Molecules Associated with Plant Maturity in Tetraploid Cultivated Potatoes (Solanum tuberosum L.)
Source: Plants (Basel). 2022 Jun 28;11(13):1707. doi: 10.3390/plants11131707 (PMC9268856; doi:10.3390/plants11131707)
Supplement: Supplementary file 1 [file plants-11-01707-s001.zip › Figure S5. KEGG pathway enrichment scatter plot of differential metabolites related to potato plant early-maturity(a) and late-maturity(b) traits.pdf]

Figure S5. KEGG pathway enrichment scatter plot of differential metabolites related to potato plant early-maturity(a) and late-maturity(b) traits

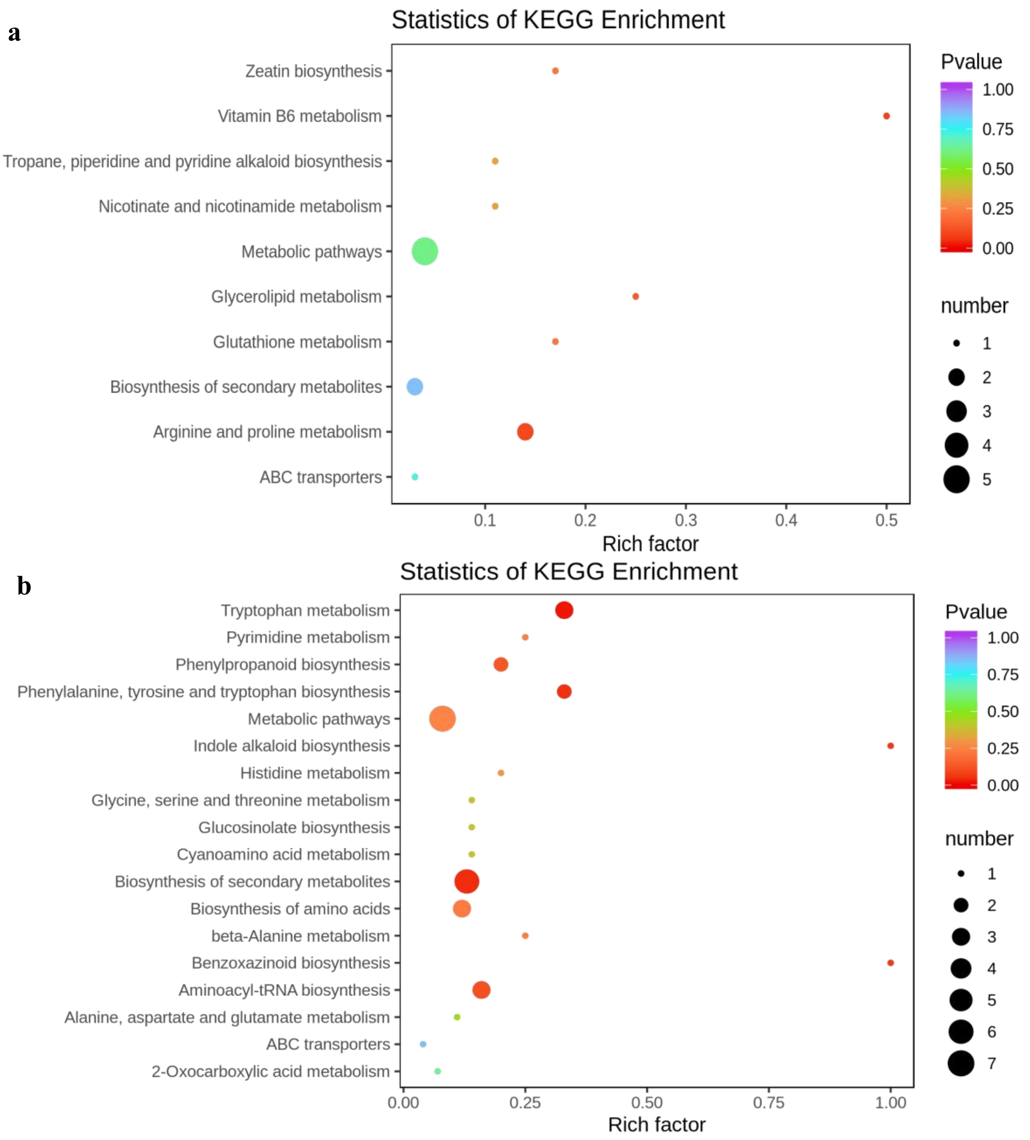

Figure S5. KEGG pathway enrichment scatter plot of differential metabolites related to potato plant early-maturity(a) and late-maturity(b) traits
