# Supplementary material for: Identification of Long-Distance Transport Signal Molecules Associated with Plant Maturity in Tetraploid Cultivated Potatoes (Solanum tuberosum L.)
Source: Plants (Basel). 2022 Jun 28;11(13):1707. doi: 10.3390/plants11131707 (PMC9268856; doi:10.3390/plants11131707)
Supplement: Supplementary file 1 [file plants-11-01707-s001.zip › Table S1. The long-distance transport mRNAs associated with potato early-maturity characteristics.pdf]

Table S1. The long-distance transport mRNAs associated with potato early-maturity characteristics

Table S1. The long-distance transport mRNAs associated with potato early-maturity characteristics

Gene ID	Chr	Gene name	Gene function
PGSC0003DMG400003104	Chr01	Nbs-lrr resistance protein	Disease-resistant protein RGA3 (A)
PGSC0003DMG400004916	Chr04	DNA binding protein	The DNA-binding protein
PGSC0003DMG400004968	Chr04	Conserved gene of unknown function	Unknown gene
PGSC0003DMG400004993	Chr12	Acetylglucosaminyltransferase	Acetaminosyltransferase
PGSC0003DMG400005630	Chr03	Non-specific lipid-transfer protein	Non-specific lipid-transferers
PGSC0003DMG400005631	Chr03	AP2/ERF transcription factor	The AP2/ERF transcription factor
PGSC0003DMG400005840	Chr08	Calcineurin B	Calcium modulation phosphatase B
PGSC0003DMG400006255	Chr09	Verticillium wilt disease resistance protein	Malwilt disease resistant protein
PGSC0003DMG400007200	Chr10	VQ	Calmodulin-binding protein
PGSC0003DMG400009480	Chr04	Sulfate transporter	Sulfate transferin
PGSC0003DMG400009941	Chr04	Receptor protein kinase CLAVATA1	CLAVATA1 receptor protein kinase
PGSC0003DMG400009995	Chr04	Phosphate transporter	Phosphate transferin
PGSC0003DMG400010020	Chr02	Autoinhibited calcium ATPase	Self-inhibition of the calcium ATP enzyme
PGSC0003DMG400011538	Chr12	Amino acid transporter	Amino acid transferin
PGSC0003DMG400011639	Chr05	Amino acid transporter	Amino acid transferin
PGSC0003DMG400011771	Chr06	Amino acid transporter	Amino acid transferin
PGSC0003DMG400012578	Chr01	UDP-glucuronosyltransferase	Uridinediphosphate glucuronate-transferase
PGSC0003DMG400014309	Chr03	StCBF2 transcription factor	The StCBF2 transcription factor
PGSC0003DMG400014310	Chr03	StCBF1 transcription factor	The StCBF1 transcription factor
PGSC0003DMG400017312	Chr07	Zinc finger family protein	Zinc finger protein
PGSC0003DMG400018884	Chr10	Ccd1	Calcium-binding protein KIC
PGSC0003DMG400019293	Chr07	NAC domain-containing protein	NAC domain protein
PGSC0003DMG400019757	Chr03	Conserved gene of unknown function	Unknown gene
PGSC0003DMG400020119	Chr06	Gene of unknown function	Unknown gene
PGSC0003DMG400022764	Chr01	Glutamate decarboxylase isoform3	Gluctamine Decarboxylase
PGSC0003DMG400023141	Chr09	Conserved gene of unknown function	Unknown gene
PGSC0003DMG400026199	Chr07	Beta-amylase	B amylase
PGSC0003DMG400026647	Chr09	Protein tyrosine kinase	Protein tyrosine kinase
PGSC0003DMG400028221	Chr10	Tropinone reductase I	Topinone reductase I
PGSC0003DMG400028358	Chr05	StMADS18 transcription factor	Promote senescence, maturity, and dormancy

PGSC0003DMG400028421	Chr02	Ubiquitin-protein ligase	ubiquitin ligase
PGSC0003DMG400029063	Chr01	Gibberellin 3beta-hydroxylase3	Akamycin 3 β hydroxylase 3
PGSC0003DMG400030713	Chr07	1,2-diacylglycerol3beta-galactosyltransferase	1,2-glyceride-3-β galactose-Asyltransferase
PGSC0003DMG400030809	Chr04	Phi-1 protein	Phi-1 protein
PGSC0003DMG400031079	Chr11	Nodulin family protein	Nodar family proteins
PGSC0003DMG400031742	Chr03	StSWEET10c	Glycemic transporters promote aging
PGSC0003DMG400033047	Chr06	StFRO7	Iron reductase gene 7
PGSC0003DMG400033693	Chr03	StSWEET11	Glycemic transporters promote aging
PGSC0003DMG400033930	Chr01	Gene of unknown function	Unknown gene
PGSC0003DMG400038115	Chr01	Gag-pol protein	Gag-pol protein
PGSC0003DMG402014665	Chr01	Gene of unknown function	Unknown gene
PGSC0003DMG402028784	Chr06	Conserved gene of unknown function	Unknown gene
PGSC0003DMG402030595	Chr02	Conserved gene of unknown function	Unknown gene
