# Supplementary material for: Identification of Long-Distance Transport Signal Molecules Associated with Plant Maturity in Tetraploid Cultivated Potatoes (Solanum tuberosum L.)
Source: Plants (Basel). 2022 Jun 28;11(13):1707. doi: 10.3390/plants11131707 (PMC9268856; doi:10.3390/plants11131707)
Supplement: Supplementary file 1 [file plants-11-01707-s001.zip › Table S2. The long-distance transport mRNAs associated with potato late-maturity characteristics.pdf]

Table S2. The long-distance transport mRNAs associated with potato late-maturity characteristics

Table S2. The long-distance transport mRNAs associated with potato late-maturity characteristics

Gene ID	Chr	Gene name	Gene function
PGSC0003DMG400000045	Chr01	Water channel protein	probable aquaporin PIP1-5 isoform X1
PGSC0003DMG400000205	Chr01	Lanatoside 15'-O-acetylesterase	GDSL esterase/lipase At4g01130-like
PGSC0003DMG400000470	Chr11	Conserved gene of unknown function	rho GTPase-activating protein gacU-like
PGSC0003DMG400000678	Chr07	Metallocarboxypeptidase inhibitor	fruit-specific protein-like
PGSC0003DMG400000932	Chr07	Reticulon family protein	reticulon-like protein B8 isoform X1
PGSC0003DMG400001089	Chr12	Conserved gene of unknown function	hypothetical protein CQW23_07395
PGSC0003DMG400001183	Chr02	Glycosyltransferase family GT8 protein	probable galacturonosyltransferase-like 1
PGSC0003DMG400001232	Chr06	Phototropic-responsive NPH3 family protein	BTB/POZ domain-containing protein At5g48800-like
PGSC0003DMG400001344	Chr02	Conserved gene of unknown function	uncharacterized protein LOC102602423
PGSC0003DMG400001465	Chr02	Protein COBRA	COBRA-like protein 4
PGSC0003DMG400001754	Chr09	Arabinogalactan protein 3	fasciclin-like arabinogalactan protein 12
PGSC0003DMG400001760	Chr09	P70 protein	interactor of constitutive active ROPs 2, chloroplastic isoform X1
PGSC0003DMG400001769	Chr09	Beta-1,3-glucuronyltransferase	probable beta-1,4-xylosyltransferase IRX9
PGSC0003DMG400001812	Chr01	Ring finger protein	RING-H2 finger protein ATL51-like
PGSC0003DMG400002040	Chr12	Hydroxyproline-rich glycoprotein	uncharacterized protein At5g65660-like
PGSC0003DMG400002074	Chr02	Pectinesterase	pectinesterase-like isoform X1
PGSC0003DMG400002085	Chr02	Pectinesterase	pectinesterase-like
PGSC0003DMG400002125	Chr04	DNA binding protein	transcription factor bHLH96-like
PGSC0003DMG400002135	Chr04	Zinc finger protein	E3 ubiquitin-protein ligase ATL15-like
PGSC0003DMG400002690	Chr09	Pyruvate kinase	pyruvate kinase 1, cytosolic
PGSC0003DMG400002895	Chr12	Sucrose synthase	sucrose synthase isoform X1
PGSC0003DMG400003063	Chr08	Gene of unknown function	remorin-like
PGSC0003DMG400003124	Chr01	Endo-1,4-beta-glucanase	endoglucanase 24-like
PGSC0003DMG400003289	Chr01	P-coumarate 3-hydroxylase	cytochrome P450 98A2-like
PGSC0003DMG400003371	Chr06	Arabinogalactan protein 2	fasciclin-like arabinogalactan protein 11
PGSC0003DMG400003639	Chr02	Conserved gene of unknown function	WEB family protein At2g17940 isoform X1
PGSC0003DMG400003678	Chr02	Nucleic acid binding protein	E3 ubiquitin-protein ligase RNF13-like
PGSC0003DMG400003691	Chr04	Phenylcoumaran benzylic ether reductase	isoflavone reductase-like protein
PGSC0003DMG400003774	Chr04	Endo-1,4-beta-glucanase	endoglucanase 10
PGSC0003DMG400003822	Chr09	Cellulose synthase	cellulose synthase, partial
PGSC0003DMG400003903	Chr09	ATSMC2	myosin-11-like
PGSC0003DMG400004118	Chr05	Auxin-induced protein 5NG4	WAT1-related protein At5g40240-like

PGSC0003DMG400004123	Chr05	Serine/threonine-protein kinase PBS1	serine/threonine-protein kinase PBS1
PGSC0003DMG400004158	Chr06	Ca2+ antiporter/cation exchanger	vacuolar cation/proton exchanger 3-like
PGSC0003DMG400004161	Chr06	Conserved gene of unknown function	protein PMR5
PGSC0003DMG400004416	Chr09	Conserved gene of unknown function	uncharacterized protein LOC107060435
PGSC0003DMG400004572	Chr12	Adenosylhomocysteinase	adenosylhomocysteinase
PGSC0003DMG400004660	Chr12	Serine hydroxymethyltransferase	serine hydroxymethyltransferase 4-like
PGSC0003DMG400004794	Chr08	Conserved gene of unknown function	uncharacterized protein LOC102579011
PGSC0003DMG400004962	Chr04	Alpha-tubulin	alpha tubulin1 [Zea mays]
PGSC0003DMG400005041	Chr03	D6-type cyclin	putative cyclin-D6-1
PGSC0003DMG400005137	Chr04	Conserved gene of unknown function	uncharacterized protein LOC102587116
PGSC0003DMG400005216	Chr01	Galactinol synthase	galactinol synthase 1
PGSC0003DMG400005273	Chr05	Peroxidase	peroxidase 3-like
PGSC0003DMG400005409	Chr01	Aspartic proteinase nepenthesin-1	protein ASPARTIC PROTEASE IN GUARD CELL 2
PGSC0003DMG400005501	Chr10	NAC domain-containing protein	NAC transcription factor 25-like
PGSC0003DMG400005671	Chr03	Beta tubulin	tubulin beta chain-like
PGSC0003DMG400005807	Chr08	NAC domain protein	NAC domain-containing protein 66-like
PGSC0003DMG400005943	Chr06	Hydrolase, hydrolyzing O-glycosyl compounds	beta-glucosidase BoGH3B-like
PGSC0003DMG400006159	Chr07	Dopamine beta-monooxygenase	cytochrome b561 and DOMON domain-containing protein At2g04850
PGSC0003DMG400006160	Chr07	Fasciclin-like arabinogalactan protein 2	fasciclin-like arabinogalactan protein 7
PGSC0003DMG400006161	Chr07	Conserved gene of unknown function	CSC1-like protein At3g21620
PGSC0003DMG400006183	Chr01	Aquaporin	aquaporin PIP2-7
PGSC0003DMG400006214	Chr10	Caffeoyl-CoA O-methyltransferase 5	O-methyltransferase, family 3
PGSC0003DMG400006497	Chr09	Conserved gene of unknown function	protein trichome birefringence-like 34 isoform X2
PGSC0003DMG400006867	Chr01	Conserved gene of unknown function	uncharacterized protein LOC102592662
PGSC0003DMG400006868	Chr01	MYB20	transcription factor MYB46
PGSC0003DMG400006880	Chr01	DRT100	DNA-damage-repair/toleration protein DRT100-like
PGSC0003DMG400007090	Chr07	Receptor protein kinase	probable receptor-like protein kinase At1g33260
PGSC0003DMG400007294	Chr06	Multidrug/pheromone exporter, MDR family, ABC transporter family	ABC transporter B family member 21-like
PGSC0003DMG400007360	Chr11	MYB20	transcription factor MYB46
PGSC0003DMG400007562	Chr03	SBT2 protein	subtilisin-like protease SBT1.7
PGSC0003DMG400007689	Chr10	Dopamine beta-monooxygenase	cytochrome b561 and DOMON domain-containing protein At3g61750
PGSC0003DMG400007902	Chr12	DNA-3-methyladenine glycosylase	probable GMP synthase [glutamine-hydrolyzing]
PGSC0003DMG400008016	Chr04	Glycogenin	UDP-glucuronate:xylan alpha-glucuronosyltransferase 2-like

			isoform X1
PGSC0003DMG400008047	Chr11	Electron transporter	uncharacterized protein LOC102592731
PGSC0003DMG400008063	Chr11	Fasciclin-like AGP 13	fasciclin-like arabinogalactan protein 11
PGSC0003DMG400008077	Chr11	Conserved gene of unknown function	uncharacterized protein LOC102590252
PGSC0003DMG400008085	Chr11	UDP-glucuronic acid/UDP-N-acetylgalactosamine transporter	uncharacterized membrane protein At1g06890
PGSC0003DMG400008090	Chr11	Kinase	receptor-like serine/threonine-protein kinase NCRK
PGSC0003DMG400008124	Chr11	Structural constituent of cell wall	arabinogalactan protein 1-like isoform X2
PGSC0003DMG400008131	Chr10	Acetylglucosaminyltransferase	beta-glucuronosyltransferase GlcAT14A
PGSC0003DMG400008403	Chr10	ATP binding / kinase/ protein serine / threonine kinase	probable leucine-rich repeat receptor-like protein kinase At5g49770
PGSC0003DMG400008598	Chr10	Conserved gene of unknown function	uncharacterized protein LOC101253847
PGSC0003DMG400008990	Chr01	Conserved gene of unknown function	protein trichome birefringence-like 11
PGSC0003DMG400009036	Chr01	Beta-glucosidase	probable beta-D-xylosidase 2
PGSC0003DMG400009086	Chr03	SF16	protein IQ-DOMAIN 1
PGSC0003DMG400009174	Chr03	SAUR family protein	auxin-responsive protein SAUR71-like
PGSC0003DMG400009381	Chr07	NAC domain protein	NAC domain-containing protein 73
PGSC0003DMG400009508	Chr03	Gene of unknown function	uncharacterized protein LOC107057941
PGSC0003DMG400009783	Chr10	Proline-rich cell wall protein	vegetative cell wall protein gp1-like
PGSC0003DMG400009915	Chr04	Zinc finger protein	protein S-acyltransferase 21 isoform X1
PGSC0003DMG400010055	Chr02	Receptor protein kinase	protein NSP-INTERACTING KINASE 1
PGSC0003DMG400010075	Chr02	Zinc finger protein	dof zinc finger protein DOF3.4
PGSC0003DMG400010545	Chr10	Conserved gene of unknown function	protein trichome birefringence-like 39
PGSC0003DMG400010726	Chr02	Rac-like GTP-binding protein RAC13	rac-like GTP-binding protein RAC13 isoform X1
PGSC0003DMG400011019	Chr10	Diphenol oxidase	laccase-4-like
PGSC0003DMG400011102	Chr10	Transcription factor HBP-1b	transcription factor TGA6-like isoform X1
PGSC0003DMG400011148	Chr07	Cellulose synthase 3	cellulose synthase A catalytic subunit 7 [UDP-forming]
PGSC0003DMG400011178	Chr07	Reticulon B21	reticulon-like protein B21 isoform X1
PGSC0003DMG400011263	Chr10	Conserved gene of unknown function	uncharacterized protein LOC102583545
PGSC0003DMG400011268	Chr10	NAC domain class transcription factor	NAC domain-containing protein 43
PGSC0003DMG400011286	Chr10	Conserved gene of unknown function	uncharacterized protein LOC102598072
PGSC0003DMG400011311	Chr01	Epoxide hydrolase 1	bifunctional epoxide hydrolase 2-like
PGSC0003DMG400011369	Chr09	Rapid alkalinization factor preproprotein	rapid alkalinization factor
PGSC0003DMG400011576	Chr09	TMS membrane family protein	probable serine incorporator
PGSC0003DMG400011746	Chr07	Pectinesterase	probable pectinesterase 8
PGSC0003DMG400012113	Chr08	NAC domain protein	NAC domain-containing protein 43
PGSC0003DMG400012170	Chr08	Dirigent protein	disease resistance response protein 206-like
PGSC0003DMG400012183	Chr08	Endo-1,4-beta-glucanase	endoglucanase 18-like

PGSC0003DMG400012221	Chr08	ATP binding protein	cingulin isoform X1
PGSC0003DMG400012458	Chr07	Disease resistance protein (TIR class)	uncharacterized protein LOC102605866
PGSC0003DMG400012756	Chr01	Gibberellin receptor GID1	probable carboxylesterase 9
PGSC0003DMG400012852	Chr12	Cellulose synthase CslG	cellulose synthase-like protein G1
PGSC0003DMG400012931	Chr03	Conserved gene of unknown function	uncharacterized protein LOC102597710 isoform X1
PGSC0003DMG400013085	Chr01	Gene of unknown function	--
PGSC0003DMG400013274	Chr11	Conserved gene of unknown function	uncharacterized protein LOC102598521
PGSC0003DMG400013439	Chr03	Aspartic proteinase oryzasin-1	aspartic proteinase A1-like
PGSC0003DMG400013547	Chr07	Sucrose sythase	sucrose synthase-like
PGSC0003DMG400013550	Chr07	Respiratory burst oxidase	respiratory burst oxidase homolog protein E-like isoform X2
PGSC0003DMG400013590	Chr11	Serine/threonine-protein kinase Nek4	serine/threonine-protein kinase Nek6-like
PGSC0003DMG400014173	Chr03	Polyphosphoinositide binding protein	random slug protein 5
PGSC0003DMG400014200	Chr03	Flotillin-1	flotillin-like protein 3
PGSC0003DMG400014250	Chr03	Serine carboxypeptidase	serine carboxypeptidase-like 45 isoform X2
PGSC0003DMG400014401	Chr10	Glycosyltransferase, CAZy family GT8	probable galacturonosyltransferase 12
PGSC0003DMG400014894	Chr02	Membrane protein	uncharacterized protein LOC102601548
PGSC0003DMG400014992	Chr01	ATP-dependent helicase	SNF2 domain-containing protein CLASSY 4-like
PGSC0003DMG400015004	Chr04	Myosin heavy chain, clone	microtubule-associated protein 70-5-like
PGSC0003DMG400015116	Chr09	Conserved gene of unknown function	protein WVD2-like 1
PGSC0003DMG400015302	Chr03	Conserved gene of unknown function	probable methyltransferase At1g27930
PGSC0003DMG400015332	Chr12	Calmodulin binding protein	protein IQ-DOMAIN 1
PGSC0003DMG400015636	Chr07	Conserved gene of unknown function	uncharacterized protein LOC102578795 isoform X2
PGSC0003DMG400015658	Chr06	Conserved gene of unknown function	uncharacterized protein LOC102579472
PGSC0003DMG400015772	Chr02	ORFX	Cell number regulator 1
PGSC0003DMG400016038	Chr07	Polygalacturonase	probable polygalacturonase
PGSC0003DMG400016053	Chr08	IQ-domain 13	protein IQ-DOMAIN 14-like
PGSC0003DMG400016167	Chr11	Wiscott-Aldrich syndrome, C-terminal	CRIB domain-containing protein RIC4-like
PGSC0003DMG400016168	Chr11	Methylenetetrahydrofolate reductase	methylenetetrahydrofolate reductase 2-like
PGSC0003DMG400016240	Chr11	Beta-hexosaminidase	beta-hexosaminidase 3
PGSC0003DMG400016302	Chr06	Class IV chitinase	endochitinase PR4-like
PGSC0003DMG400016488	Chr02	Conserved gene of unknown function	uncharacterized protein LOC102599053
PGSC0003DMG400016825	Chr10	Axi 1 protein	uncharacterized protein At1g04910-like
PGSC0003DMG400017178	Chr09	Rhicadhesin receptor	germin-like protein subfamily 1 member 17
PGSC0003DMG400017199	Chr09	ARPC2B	actin-related protein 2/3 complex subunit 2B isoform X1
PGSC0003DMG400017592	Chr10	P70 protein	interactor of constitutive active ROPs 2,

			chloroplastic-like isoform X2
PGSC0003DMG400017761	Chr12	Ring finger protein	RING-H2 finger protein ATL54
PGSC0003DMG400017764	Chr12	Auxin-independent growth promoter	uncharacterized protein At1g04910-like
PGSC0003DMG400017931	Chr06	Conserved gene of unknown function	uncharacterized protein LOC102586733
PGSC0003DMG400018174	Chr03	Short-chain dehydrogenase	short-chain dehydrogenase TIC 32, chloroplastic-like
PGSC0003DMG400018658	Chr01	Axi 1 protein	uncharacterized protein At1g04910-like
PGSC0003DMG400018849	Chr03	Leucine-rich repeat receptor kinase	leucine-rich repeat receptor-like protein kinase TDR
PGSC0003DMG400018877	Chr10	Aspartic proteinase nepenthesin-1	protein ASPARTIC PROTEASE IN GUARD CELL 2-like
PGSC0003DMG400018887	Chr10	Conserved gene of unknown function	uncharacterized protein LOC102600644
PGSC0003DMG400018930	Chr01	Proteinase inhibitor I4, serpin	transmembrane protein 45B-like
PGSC0003DMG400019139	Chr10	Rop guanine nucleotide exchange factor	rop guanine nucleotide exchange factor 7-like
PGSC0003DMG400019175	Chr10	Conserved gene of unknown function	protein trichome birefringence-like 3
PGSC0003DMG400019185	Chr10	Diphenol oxidase	laccase-4-like
PGSC0003DMG400019228	Chr07	Glycosyltransferase, CAZy family GT8	probable galacturonosyltransferase 12
PGSC0003DMG400019243	Chr07	Conserved gene of unknown function	calcium-binding protein PBP1
PGSC0003DMG400019264	Chr07	Kinesin	kinesin-3-like
PGSC0003DMG400019350	Chr09	ATP binding protein	serine/threonine-protein kinase Nek2
PGSC0003DMG400019656	Chr05	GMP synthase	DNA-3-methyladenine glycosylase 1
PGSC0003DMG400020160	Chr06	Cytochrome b5	cytochrome b5-like
PGSC0003DMG400020204	Chr02	Hydrolase, acting on ester bonds	pollen allergen Che a 1
PGSC0003DMG400020258	Chr02	Blue copper protein	mavicyanin-like
PGSC0003DMG400020345	Chr09	Diphenol oxidase	laccase-4
PGSC0003DMG400020355	Chr09	Conserved gene of unknown function	laccase-17-like
PGSC0003DMG400020364	Chr09	Receptor protein kinase	probable serine/threonine-protein kinase At5g41260
PGSC0003DMG400020438	Chr07	Serine/threonine-protein kinase PBS1	serine/threonine-protein kinase CDL1
PGSC0003DMG400020545	Chr06	BCL-2 binding anthanogene-1	BAG family molecular chaperone regulator 4-like
PGSC0003DMG400021033	Chr06	UDP-glucuronic acid/UDP-N-acetylgalactosamine transporter	uncharacterized membrane protein At1g06890-like
PGSC0003DMG400021404	Chr02	Conserved gene of unknown function	hypothetical protein A4A49_38207
PGSC0003DMG400021537	Chr04	Conserved gene of unknown function	uncharacterized protein LOC102578171
PGSC0003DMG400021576	Chr06	NAC domain-containing protein	NAC domain-containing protein 7-like
PGSC0003DMG400021577	Chr06	NAC domain protein	NAC domain-containing protein 7-like
PGSC0003DMG400021728	Chr02	Conserved gene of unknown function	uncharacterized protein LOC102604949 isoform X1
PGSC0003DMG400021866	Chr10	Aminotransferase family protein	probable aminotransferase TAT2
PGSC0003DMG400022097	Chr07	Conserved gene of unknown function	uncharacterized protein LOC102578328
PGSC0003DMG400022153	Chr07	Conserved gene of unknown function	uncharacterized protein LOC102600019

PGSC0003DMG400022182	Chr07	Rhicadhesin receptor	germin-like protein 11-1
PGSC0003DMG400022365	Chr02	Beta-galactosidase	beta-galactosidase 8-like
PGSC0003DMG400022533	Chr03	Conserved gene of unknown function	WEB family protein At2g17940-like
PGSC0003DMG400022636	Chr05	Germin 5	germin-like protein subfamily 1 member 1
PGSC0003DMG400022646	Chr05	Conserved gene of unknown function	hypothetical protein A4A49_61894
PGSC0003DMG400022674	Chr01	Glutaredoxin	monothiol glutaredoxin-S3
PGSC0003DMG400022725	Chr08	Subtilisin-like protease	subtilisin-like protease SBT1.7
PGSC0003DMG400022904	Chr03	Conserved gene of unknown function	uncharacterized protein LOC102603463
PGSC0003DMG400022905	Chr03	Multidrug/pheromone exporter, MDR family, ABC transporter family	ABC transporter B family member 19-like
PGSC0003DMG400022964	Chr02	Lanceolate	transcription factor TCP10-like
PGSC0003DMG400022965	Chr02	Calmodulin binding protein	protein IQ-DOMAIN 1-like
PGSC0003DMG400023205	Chr10	Amino acid transporter	LAX5 protein
PGSC0003DMG400023324	Chr05	Aspartic proteinase nepenthesin-1	aspartic proteinase nepenthesin-1
PGSC0003DMG400023361	Chr05	Methylenetetrahydrofolate reductase	probable methylenetetrahydrofolate reductase
PGSC0003DMG400023458	Chr05	Phenylalanine ammonia-lyase	phenylalanine ammonia-lyase-like
PGSC0003DMG400023483	Chr05	Self-pruning interacting protein 1	cyclin-dependent protein kinase inhibitor SMR1
PGSC0003DMG400023524	Chr05	UDP-glucuronate decarboxylase 1	UDP-glucuronic acid decarboxylase 6-like
PGSC0003DMG400023570	Chr04	Conserved gene of unknown function	protein ODORANT1-like
PGSC0003DMG400023699	Chr10	Patatin B2	patatin-like protein 3
PGSC0003DMG400023707	Chr10	Homeobox-leucine zipper protein HAT14	homeobox-leucine zipper protein HOX11-like isoform X1
PGSC0003DMG400023740	Chr10	Gene of unknown function	hypothetical protein T459_26476
PGSC0003DMG400023940	Chr06	Conserved gene of unknown function	protein trichome birefringence-like 33
PGSC0003DMG400024237	Chr06	Conserved gene of unknown function	protein ESKIMO 1-like
PGSC0003DMG400024286	Chr09	Ca2+ antiporter/cation exchanger	cation/calcium exchanger 1-like
PGSC0003DMG400024289	Chr09	Reticuline oxidase	hypothetical protein BC332_00887
PGSC0003DMG400024474	Chr03	Gonadotropin beta chain	gibberellin-regulated protein 10-like
PGSC0003DMG400024530	Chr03	Protein COBRA	COBRA-like protein 4
PGSC0003DMG400024550	Chr03	ZPT4-3	zinc finger protein ZAT1-like
PGSC0003DMG400024664	Chr03	Conserved gene of unknown function	uncharacterized protein LOC107060149
PGSC0003DMG400024897	Chr01	Conserved gene of unknown function	WEB family protein At3g51220
PGSC0003DMG400024989	Chr02	Nucleic acid binding protein	protein IRX15-LIKE-like
PGSC0003DMG400025104	Chr05	AGP19 (ARABINOGALACTAN-PROTEIN 19)	lysine-rich arabinogalactan protein 19
PGSC0003DMG400025346	Chr03	Voltage-dependent anion channel	mitochondrial outer membrane protein porin of 36 kDa-like
PGSC0003DMG400025474	Chr02	Nucleic acid binding protein	protein IRX15-LIKE-like
PGSC0003DMG400025579	Chr06	Conserved gene of unknown function	probable beta-1,4-xylosyltransferase IRX10L

PGSC0003DMG400025771	Chr01	Interleukin-1 receptor-associated kinase	probable LRR receptor-like serine/threonine-protein kinase At4g37250
PGSC0003DMG400025882	Chr01	Caffeoyl-CoA O-methyltransferase 6	O-methyltransferase, family 3
PGSC0003DMG400025985	Chr01	Conserved gene of unknown function	uncharacterized protein LOC102594113
PGSC0003DMG400026004	Chr01	Leucine-rich repeat receptor kinase	LRR receptor-like serine/threonine-protein kinase GSO1
PGSC0003DMG400026113	Chr06	Conserved gene of unknown function	uncharacterized protein LOC102593915
PGSC0003DMG400026189	Chr07	Xyloglucan endotransglycosylase/hydrolase 16 protein	xyloglucan endotransglucosylase/hydrolase protein 9
PGSC0003DMG400026463	Chr12	Aquaporin TIP2	aquaporin TIP2-1
PGSC0003DMG400026516	Chr06	Conserved gene of unknown function	uncharacterized protein At1g04910-like isoform X1
PGSC0003DMG400026760	Chr04	Receptor-kinase	inactive leucine-rich repeat receptor-like serine/threonine-protein kinase At1g60630
PGSC0003DMG400026837	Chr12	Profilin-1	profilin-1
PGSC0003DMG400026944	Chr06	UPF0497 membrane protein 8	CASP-like protein 1F1
PGSC0003DMG400027037	Chr06	ATP binding protein	WEB family protein At3g02930, chloroplastic-like
PGSC0003DMG400027079	Chr06	Alliin lyase	tryptophan aminotransferase-related protein 2-like
PGSC0003DMG400027168	Chr05	Laccase 90a	laccase-12-like
PGSC0003DMG400027219	Chr05	Conserved gene of unknown function	protein trichome birefringence-like 33
PGSC0003DMG400027681	Chr06	Dopamine beta-monooxygenase	cytochrome b561 and DOMON domain-containing protein At3g07570-like
PGSC0003DMG400027728	Chr03	Conserved gene of unknown function	protein ESKIMO 1
PGSC0003DMG400027977	Chr06	SKIP interacting protein 19	BTB/POZ domain-containing protein At3g56230
PGSC0003DMG400028126	Chr10	Penetration and arbuscule morphogenesis protein	ankyrin-1-like
PGSC0003DMG400028207	Chr10	Peptidase C14, caspase catalytic subunit p20	metacaspase-9
PGSC0003DMG400028267	Chr10	Cellulose synthase-like A1	glucomannan 4-beta-mannosyltransferase 9-like
PGSC0003DMG400028357	Chr05	Conserved gene of unknown function	uncharacterized protein LOC102586821
PGSC0003DMG400028388	Chr05	Conserved gene of unknown function	uncharacterized protein LOC102581532 isoform X2
PGSC0003DMG400028415	Chr02	Conserved gene of unknown function	uncharacterized protein LOC102580891
PGSC0003DMG400028426	Chr02	Cellulose synthase catalytic subunit	cellulose synthase A catalytic subunit 8 [UDP-forming]
PGSC0003DMG400028439	Chr02	Growth regulator	uncharacterized protein At1g04910 isoform X1
PGSC0003DMG400028578	Chr01	DNA binding protein	transcription factor bHLH94-like
PGSC0003DMG400028601	Chr01	Alpha-L-fucosidase 2	GDSL esterase/lipase At1g54790

PGSC0003DMG400028648	Chr12	Blue copper	lamin-like protein
PGSC0003DMG400028707	Chr04	Conserved gene of unknown function	uncharacterized protein LOC102579039
PGSC0003DMG400028773	Chr10	Conserved gene of unknown function	vicianin hydrolase-like
PGSC0003DMG400028801	Chr06	ATP binding protein	probable inactive receptor kinase At1g48480
PGSC0003DMG400028816	Chr12	Conserved gene of unknown function	protein JASON
PGSC0003DMG400028825	Chr12	MATE transporter	protein DETOXIFICATION 41
PGSC0003DMG400028857	Chr12	Conserved gene of unknown function	uncharacterized protein LOC102584804
PGSC0003DMG400028929	Chr06	4-coumarate--CoA ligase 2	4-coumarate--CoA ligase 2
PGSC0003DMG400029080	Chr06	LOB domain-containing protein	LOB domain-containing protein 15
PGSC0003DMG400029110	Chr05	PLE	65-kDa microtubule-associated protein 8
PGSC0003DMG400029418	Chr12	Tetracycline transporter	tetracycline resistance protein, class E-like
PGSC0003DMG400029575	Chr08	Catechol oxidase B, chloroplastic	catechol oxidase B, chloroplastic
PGSC0003DMG400029790	Chr12	Zinc finger family protein	E3 ubiquitin-protein ligase At4g11680
PGSC0003DMG400030081	Chr09	Conserved gene of unknown function	uncharacterized protein LOC102583145
PGSC0003DMG400030088	Chr09	Conserved gene of unknown function	hypothetical protein CQW23_22348
PGSC0003DMG400030172	Chr09	Aspartic proteinase oryzasin-1	aspartic proteinase oryzasin-1-like
PGSC0003DMG400030450	Chr06	Protein kinase	serine/threonine-protein kinase STY17
PGSC0003DMG400030567	Chr05	Zinc finger protein	zinc finger CCCH domain-containing protein 15-like
PGSC0003DMG400030591	Chr02	Zinc finger protein	zinc finger CCCH domain-containing protein 14-like
PGSC0003DMG400030593	Chr02	Proteinase inhibitor IIa	proteinase inhibitor type-2-like
PGSC0003DMG400030839	Chr04	Blue copper protein	blue copper protein-like
PGSC0003DMG400031044	Chr03	MYB transcription factor	transcriptional activator Myb-like isoform X2
PGSC0003DMG400031065	Chr11	Pectate lyase	probable pectate lyase 13 isoform X1
PGSC0003DMG400031183	Chr11	Conserved gene of unknown function	glucuronoxylan 4-O-methyltransferase 3
PGSC0003DMG400031364	Chr10	Adenosine kinase isoform 2S	adenosine kinase 1-like
PGSC0003DMG400031365	Chr10	Phenylalanine ammonia-lyase	Aromatic amino acid lyase
PGSC0003DMG400031385	Chr10	Pectin acetylesterase	pectin acetylesterase 10-like isoform X2
PGSC0003DMG400031770	Chr02	Conserved gene of unknown function	uncharacterized protein LOC102587035
PGSC0003DMG400031790	Chr09	Epoxide hydrolase	bifunctional epoxide hydrolase 2-like
PGSC0003DMG400031822	Chr09	Kinesin heavy chain	kinesin-4-like
PGSC0003DMG400032147	Chr01	Peroxidase	lignin-forming anionic peroxidase-like
PGSC0003DMG400032229	Chr01	Dirigent 2	dirigent protein 19
PGSC0003DMG400032828	Chr07	Serine-threonine protein kinase, plant-type	leucine-rich repeat extensin-like protein 4
PGSC0003DMG400033872	Chr05	Delta-8 sphingolipid desaturase	delta(8)-fatty-acid desaturase-like
PGSC0003DMG400034688	Chr02	Alpha tubulin 1	hypothetical protein MANES_10G087200
PGSC0003DMG400037865	Chr06	Conserved gene of unknown function	protein ESKIMO 1-like
PGSC0003DMG400041435	Chr09	Arabinogalactan protein	fasciclin-like arabinogalactan protein 12
PGSC0003DMG400047074	Chr08	BURP domain-containing protein	BURP domain-containing protein 3-like

PGSC0003DMG401004451	Chr09	Conserved gene of unknown function	protein trichome birefringence-like 3
PGSC0003DMG401008903	Chr09	Conserved gene of unknown function	laccase-17-like
PGSC0003DMG401009045	Chr01	GRAS family transcription factor	DELLA protein RGL1-like
PGSC0003DMG401009727	Chr01	Calcium ion binding protein	probable peroxygenase 5
PGSC0003DMG401013295	Chr11	Myosin heavy chain, clone	microtubule-associated protein 70-5-like
PGSC0003DMG401018597	Chr05	Alpha-L-fucosidase 2	GDSL esterase/lipase At3g26430-like
PGSC0003DMG401023679	Chr10	Kinase family protein	serine/threonine-protein kinase Nek6-like isoform X1
PGSC0003DMG401027132	Chr05	Lipase	uncharacterized protein LOC102591372
PGSC0003DMG401032203	Chr01	Extensin	RNA-binding protein 26-like isoform X2
PGSC0003DMG402002121	Chr04	Glycine-rich protein	hypothetical protein SOVF_125620
PGSC0003DMG402003586	Chr02	Cell number regulator 13	protein MID1-COMPLEMENTING ACTIVITY 1-like
PGSC0003DMG402004586	Chr12	Wiscott-Aldrich syndrome, C-terminal	CRIB domain-containing protein RIC4-like isoform X1
PGSC0003DMG402009228	Chr11	Beta-galactosidase	beta-galactosidase 16-like isoform X3
PGSC0003DMG402013464	Chr03	O-acetyltransferase	protein REDUCED WALL ACETYLATION 4-like isoform X1
PGSC0003DMG402015315	Chr12	Fiber protein Fb34	uncharacterized protein LOC102597259
PGSC0003DMG402017962	Chr01	ATP binding / microtubule motor	LOW QUALITY PROTEIN: kinesin-like protein KIFC3
PGSC0003DMG402019682	Chr11	Gene of unknown function	uncharacterized protein LOC102583250
PGSC0003DMG402024737	Chr01	Alpha-L-fucosidase 2	acetylajmalan esterase-like isoform X1
PGSC0003DMG402026034	Chr01	Oxidoreductase	2-oxoglutarate-dependent dioxygenase AOP2-like
PGSC0003DMG402027116	Chr05	Laccase 90d	laccase-12-like
PGSC0003DMG402027132	Chr05	Lipase	uncharacterized protein LOC107062158
PGSC0003DMG402032203	Chr01	Extensin	RNA-binding protein 26-like isoform X2
